# Combination Therapy with Cinnamaldehyde and Hyperthermia Induces Apoptosis of A549 Non-Small Cell Lung Carcinoma Cells via Regulation of Reactive Oxygen Species and Mitogen-Activated Protein Kinase Family

**DOI:** 10.3390/ijms21176229

**Published:** 2020-08-28

**Authors:** Jinbong Park, Seung Ho Baek

**Affiliations:** 1 Department of Pharmacology, College of Korean Medicine, Kyung Hee University, Seoul 02447, Korea; thejinbong@khu.ac.kr; 2 College of Korean Medicine, Dongguk University, 32 Dongguk-ro, Ilsandong-gu, Goyang-si, Gyeonggi-do 10326, Korea

**Keywords:** non-small cell lung cancer, cinnamaldehyde, hyperthermia therapy, synergy, apoptosis, reactive oxygen species, mitogen-activated protein kinase

## Abstract

Lung cancer is the largest cause of cancer-induced deaths. Non-small cell lung cancer (NSCLC) is the most frequently observed subtype of lung cancer. Although recent studies have provided many therapeutic options, there is still a need for effective and safe treatments. This paper reports the combined effects of cinnamaldehyde (CNM), a flavonoid from cinnamon, together with hyperthermia, a therapeutic option for cancer treatment, on the A549 NSCLC cell line. A hyperthermia treatment of 43 °C potentiated the cytotoxicity of CNM in A549 cells. This was attributed to an increase in the apoptosis markers and suppression of the survival/protective factors, as confirmed by Western blot assays. Flow cytometry supported this result because the apoptotic profile, cell health profile, and cell cycle profile were regulated by CNM and hyperthermia combination therapy. The changes in reactive oxygen species (ROS) and its downstream target pathway, mitogen-activated protein kinases (MAPK), were evaluated. The CNM and hyperthermia combination increased the generation of ROS and MAPK phosphorylation. N-acetylcysteine (NAC), a ROS inhibitor, abolished the apoptotic events caused by CNM and hyperthermia co-treatment, suggesting that the cytotoxic effect was dependent of ROS signaling. Therefore, we suggest CNM and hyperthermia combination as an effective therapeutic option for the NSCLC treatment.

## 1. Introduction

Cancer is one of the most crucial health issues worldwide. Among the 9.6 million deaths from cancer, lung cancer accounts for approximately 20% of global cancer mortality [1]. Approximately 80–85% of lung cancer patients express a histological subtype known as non-small cell lung cancer (NSCLC) [2]. Progress in treating this fatal disease has been promising over the past 20 years, but the majority of options are still limited to cytotoxic chemotherapy, despite side effects, and drug resistance [3].

Therefore, natural products have attracted interest as an alternative therapy for NSCLC. Cinnamaldehyde (CNM), the main ingredient of cinnamon, is a widely used flavoring agent [4]. Several studies have reported the beneficial effects of CNM in various types of cancers, including leukemia [5], melanoma [6], colorectal cancer [7], and lung cancer [8,9].

Hyperthermia is another potential method that may trigger cancer cell death. High-temperature stimulation leads to several physiological responses, including changes in the membrane permeability or cytoskeleton system, alteration in macromolecule synthesis or intracellular signaling, and inhibition of DNA repair [10]. In lung cancer, hyperthermia treatment induces cell death via cytoskeletal alteration [11], an increase in caspase-3-dependent apoptosis [12], or the induction of reactive oxygen species-related autophagy [13]. In addition, the effects of gemicitabine [14], paclitaxel/carboplatin [15], doxorubicin [16], cisplatin [17], or even radiotherapy [18] can be increased when combined with hyperthermia. Moreover, many studies of a combination of hyperthermia with natural products, such as beta-lapachon [19], Rhizoma paridis [20], and β-elemene [21], have been carried out to show the beneficial use of nature-derived agents when combined with hyperthermia. To date, there are no reports on the combination treatment of hyperthermia with CNM, a well-known nature-derived material with anti-cancerous effects, in NSCLC. This study evaluated the synergism between CNM and hyperthermia treatment in the NSCLC cell line, A549 cells, and investigated the underlying mechanisms.

## 2. Results

### 2.1. Combination Therapy of CNM and Hyperthermia Synergistically Inhibits Cell Proliferation of A549 Cells

**Figure 1:**
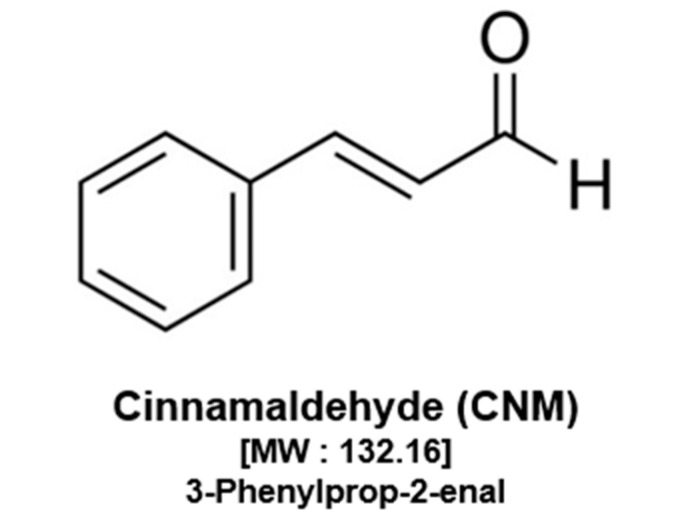
Molecular structure of cinnamaldehyde (CNM).

**Figure 2:**
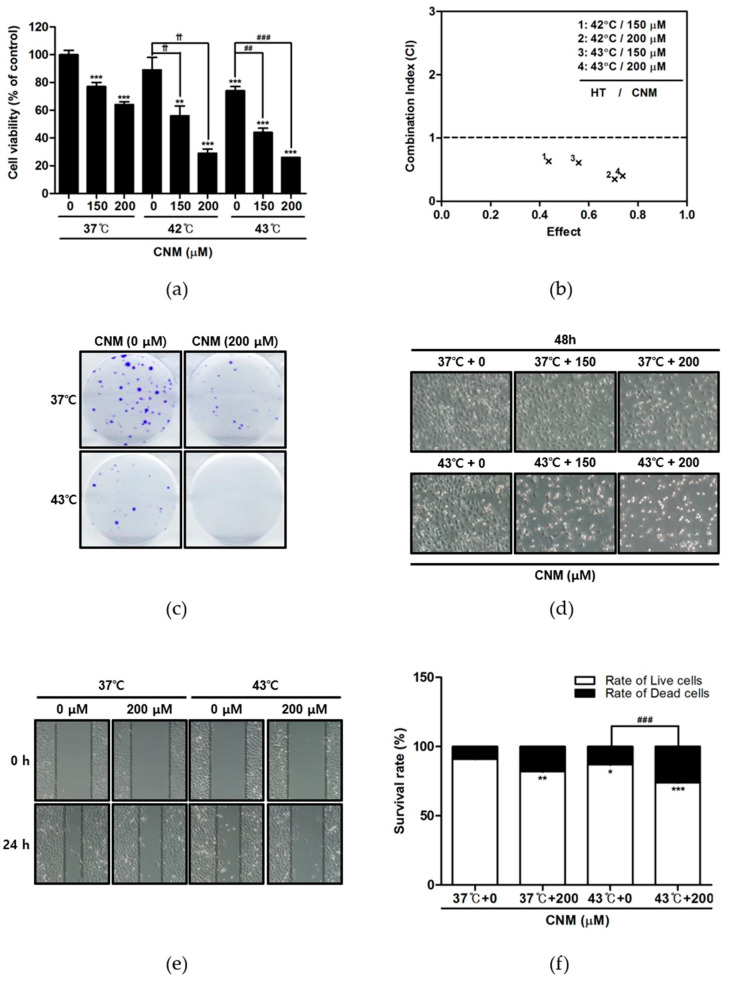
Effect of CNM and hyperthermia combination therapy on cell viability in A549 cells. A549 cells were treated with CNM (0, 150 or 200 μM) with or without hyperthermia and incubated for 24 h. (**a**) The cell viability was determined by an MTT assay. ** *p* < 0.01, *** *p* < 0.001 vs. 37 °C + 0 μM group; ^††^ *p* < 0.01 vs. 42 °C + 0 μM group; ^##^ *p* < 0.01, ^###^ *p* < 0.001 vs. 43 °C + 0 μM group; (**b**) The combination index on cytotoxicity effect was determined using CompuSyn Software; (**c**) a clonogenic assay was performed by staining cells with Crystal violet staining; (**d**) morphological changes reflecting apoptosis were visualized under a regular light microscope (magnification ×100); (**e**) wound healing assays were performed; (**f**) the live and dead cell portion was determined by Trypan blue staining. * *p* < 0.05, ** *p* < 0.01, *** *p* < 0.001 vs. control group; ^###^ *p* < 0.001 vs. 43 °C + 0 μM group.

First, the anti-proliferative effects of CNM (Figure 1) and hyperthermia co-treatment were evaluated using an MTT assay. CNM treatment in normothermia (37 °C) or hyperthermia (42 and 43 °C) condition showed a decrease in the cell viability of A549 cells. In particular, a co-treatment with hyperthermia of 42 and 43 °C inhibited cell proliferation compared to that at 37 °C (Figure 2a). We calculated the combination index using the CompuSyn software to find out CNM combined with hyperthermia (42 and 43 °C) showed combination indexes lower than 1 (Figure 2b), which indicates synergistic combination. Crystal violet staining of viable cells indicated a dramatic decrease in colony formation in A549 cells treated with a combination of CNM and 43 °C hyperthermia, while hyperthermia treatment or CNM treatment in normothermia conditions resulted in a moderate decrease (Figure 2c). Visual observation of the cell morphology also showed a decrease in the cell numbers (Figure 2d), confirming the anti-proliferative effect of CNM and hyperthermia combination. Additional wound healing assays verified the inhibition of cell migration by the co-treatment of CNM and hyperthermia (Figure 2e). Furthermore, the dead cell portion determined by a Trypan blue staining assay was increased markedly by the co-treatment therapy (Figure 2f).

### 2.2. Combination Therapy of CNM and Hyperthermia Increases Apoptosis Markers and Suppresses Survival Markers in A549 Cells

**Figure 3:**
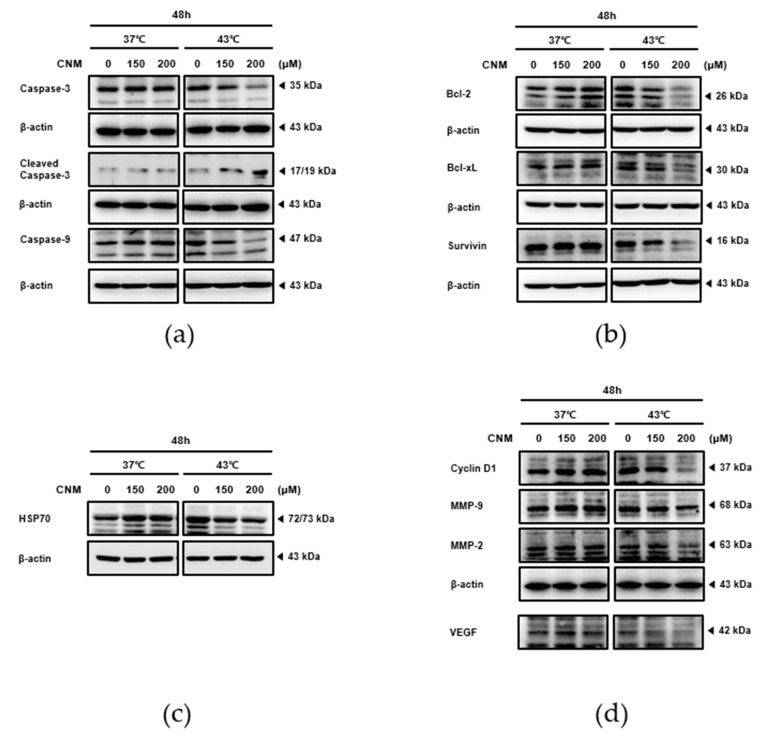
Effect of CNM and hyperthermia combination therapy on the protein levels of apoptosis and survival markers in A549 cells. A549 cells were treated with CNM (0, 150 or 200 μM) with or without hyperthermia and incubated for 24 h. Whole-cell extracts were prepared, then equal concentrations of lysates were analyzed by Western blot analysis. Protein expression of (**a**) caspase-3, caspase-9, (**b**) Bcl-2, Bcl-xL, Survivin, (**c**) HSP70, (**d**) Cyclin D1, VEGF, MMP-2 and MMP-9 was measured using Western blot assays. β-actin was used as a loading control. Representative blots are shown.

The expression levels of the factors related to apoptosis, proliferation, metastasis, and angiogenesis were next examined to verify the action mechanism of CNM and hyperthermia co-treatment. As a result, co-treatment with CNM 200 μM and hyperthermia of 43 °C induced the cleavage of caspase-3 (Figure 3a), which is the final step in programmed apoptosis [22]. On the other hand, such an effect was not observed under the 37 °C condition. Further proteins in the apoptosis pathway were investigated by additional Western blot assays. In line with the result of cleaved caspase-3, the level of caspase-9 expression decreased in a dose-dependent manner, but only by the CNM and 43 °C hyperthermia co-treatment (Figure 3a). In addition, the anti-apoptotic members of the B-cell lymphoma (Bcl)-2 family, Bcl-2, Bcl-xL, and Survivin [23], were also suppressed by the combination treatment of CNM and 43 °C (Figure 3b). Western blot assays were conducted to determine if heat shock protein 70 (HSP70) was involved in the action of CNM and hyperthermia. The results show that CNM co-treatment reversed the increase in HSP70 expression in response to hyperthermia (Figure 3c). Moreover, the CNM and hyperthermia co-treatment regulated the cell cycle while reducing the metastatic potential of A549 cells. This was illustrated by the inhibition of the expression of Cyclin D1, vascular endothelial growth factor (VEGF), matrix metallopeptidase (MMP)-2 and MMP-9 by the combination of CNM and 43 °C hyperthermia (Figure 3d).

### 2.3. Combination of CNM and Hyperthermia Induces Apoptosis by Arresting Cell Cycle in A549 Cells

**Figure 4:**
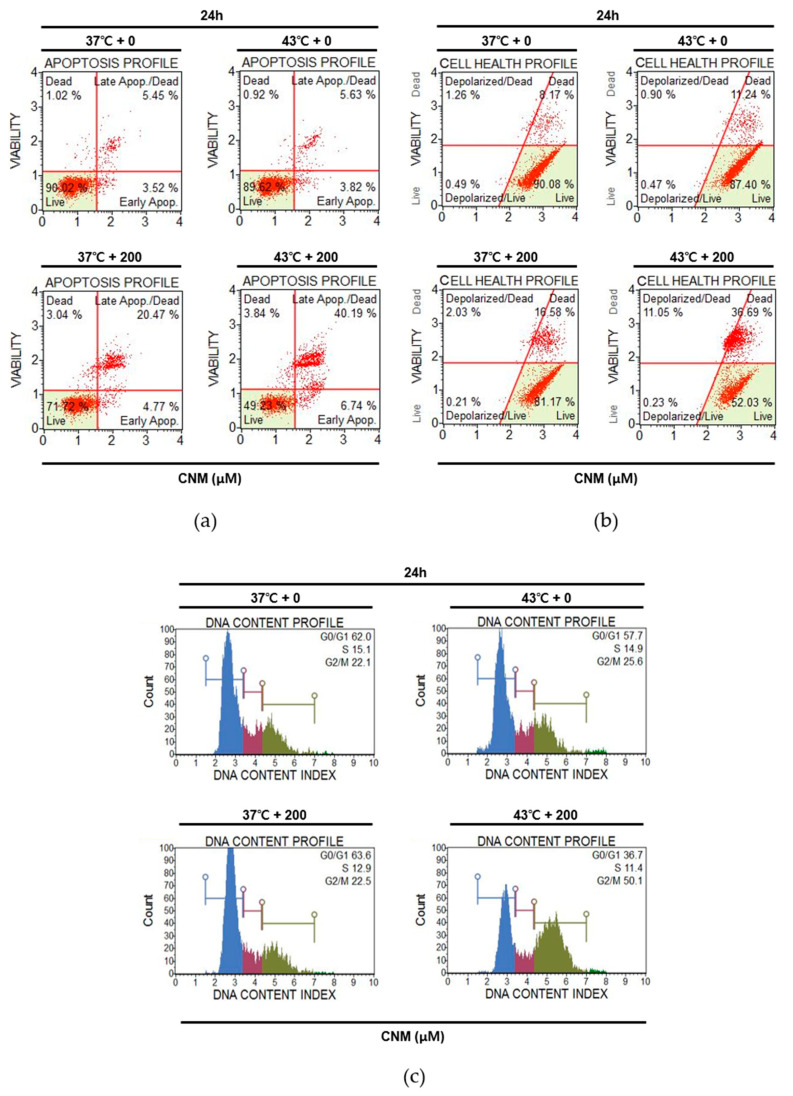
Effect of CNM and hyperthermia combination therapy on the apoptosis profile, cell health profile and cell cycle profile in A549 cells. A549 cells were treated with CNM (0, 200 μM) with or without hyperthermia and incubated for 2 h. Annexin V and 7-AAD staining was used to detect apoptosis and then analyzed by a flow cytometer. Flow cytometry analysis on (**a**) apoptosis profile, (**b**) mitochondrial membrane potential profile and (**c**) cell cycle profile was performed.

Cell cycle arrest is closely related to the induction of apoptosis and is frequently used as the therapeutic target of anti-cancer agents [24]. Flow cytometry analyses were carried out to determine if cell cycle arrest also occurs in the action mechanism of CNM and hyperthermia combination treatment. CNM with hyperthermia treatment of 43 °C increased the Annexin V-associated apoptotic profile of A549 cells. The CNM treatment at 37 °C increased the rate of apoptosis from 8.97% to 25.24%, but when combined with hyperthermia, the number of apoptotic cells grew to 46.93% of the total cells (Figure 4a). In addition, as shown in Figure 4b, when the mitochondrial membrane potential of A549 cells was measured to determine the changes in the cell health profile, the CNM treatment accompanied by 43 °C hyperthermia regulated the dead cell ratio of A549 cells after 24 h to 47.74% of the total cells compared to either a sole treatment of 43 °C hyperthermia (20.24%) or a combination of CNM and 37 °C normothermia (18.61%). Next, the cell cycle in A549 cells treated with CNM and hyperthermia was analyzed. The results indicate that cell cycle arrest was induced at the G2/M phase by a co-treatment of CNM and 43 °C hyperthermia (Figure 4c) accompanied with the decrease in Cyclin B1 (Supplementary Figure S1), a key regulator of cellular mitosis [25].

### 2.4. Combination Therapy of CNM and Hyperthermia Increases Reactive Oxygen Species (ROS) Production and Induces Its Downstream Mitogen-Activated Protein Kinase (MAPK) Pathway

**Figure 5:**
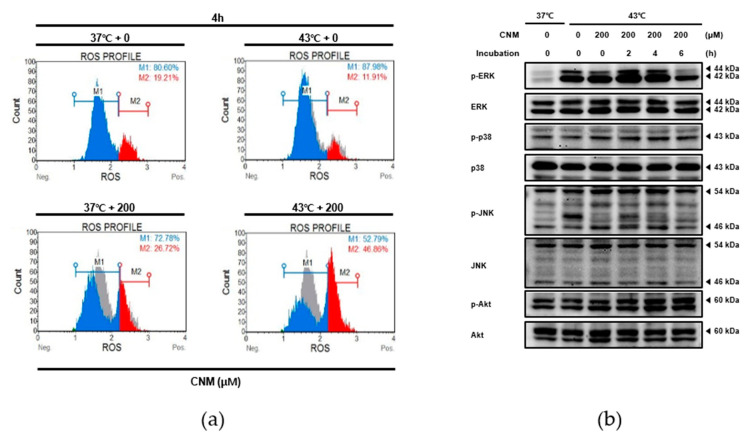
Effect of CNM and hyperthermia combination therapy on reactive oxygen species (ROS) generation and MAPK pathway in A549 cells. A549 cells were treated with CNM (0 or 200 μM) with or without hyperthermia and incubated for the indicated time. (**a**) Flow cytometry analysis on ROS generation was performed; (**b**) Protein expressions of p-ERK, ERK, p-JNK, JNK, p-p38, p38, p-Akt and Akt were measured using western blot assays. Representative blots are shown.

ROS signaling is one of the target mechanisms to induce apoptosis in cancer cells that hyperthermia and several natural products share [26,27]. Therefore, the next goal was to evaluate the role of ROS in the pro-apoptotic effect from the combination of CNM and hyperthermia treatment. When assessed by flow cytometry, ROS release was increased significantly by the CNM plus hyperthermia combination treatment (Figure 5a). The levels of MAPKs phosphorylation, which is an important downstream pathway of ROS signaling [28], were evaluated by Western blot assays. As shown in Figure 5b, increases in phosphorylation of all three MAPKs, extracellular signal-regulated kinase (ERK), c-Jun NH2-terminal kinase (JNK) and p38, along with Akt were observed in A549 cells treated with the CNM and hyperthermia combination. In particular, the peak of p-ERK was observed at 2 h post-treatment. Phosphorylation of p38 showed a similar time-dependent pattern as ERK, while p-JNK showed a gradual increase until 4 h. On the other hand, phosphorylation of Akt showed a time-dependent increase starting from right after treatment until 6 h post-treatment.

### 2.5. Apoptotic Effect by Combination Therapy of CNM and Hyperthermia in A549 Cells Is Dependent On ROS Signaling

**Figure 6:**
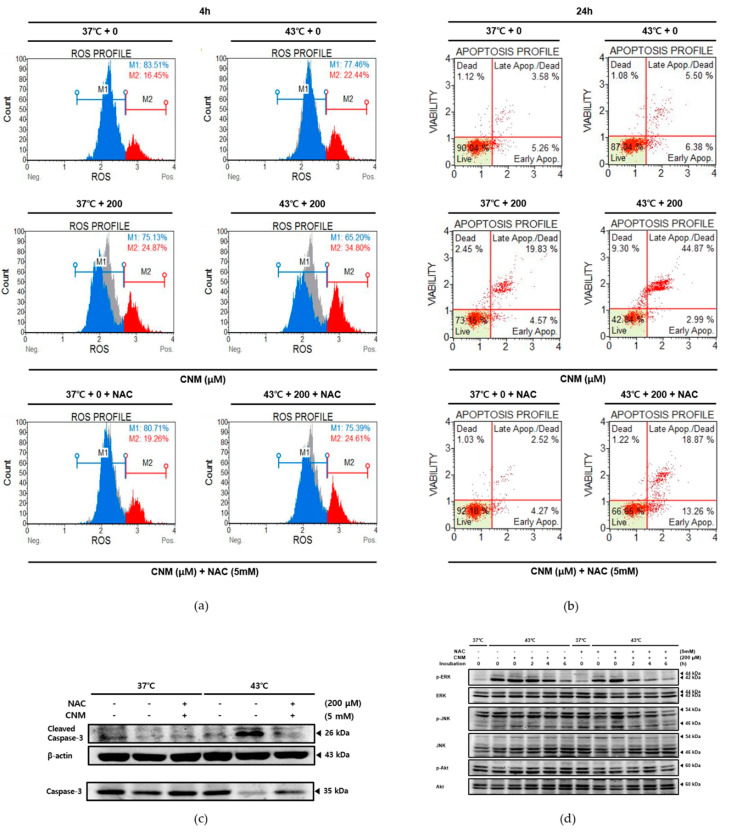
Effect of CNM and hyperthermia combination therapy on ROS generation, apoptosis markers and MAPK pathway in ROS-inhibited A549 cells. A549 cells were pre-treated with N-acetylcysteine (NAC) and then treated with CNM (0 or 200 μM) with or without hyperthermia and incubated for the indicated time. Flow cytometry analysis on (**a**) ROS generation and (**b**) apoptosis profile was performed. β-actin was used as a loading control. Protein expressions of (**c**) caspase-3, caspase-9, (**d**) p-ERK, ERK, p-JNK, JNK, p-Akt and Akt were measured using Western blot assays. Representative blots are shown. (−), absence of NAC or CNM; (+), presence of NAC or CNM.

The cells were pretreated with N-acetylcysteine (NAC), a free radical scavenger, and thus a ROS inhibitor, before the combination treatment of CNM and thermal stimulation. As shown in Figure 6a, the NAC pre-treatment blocked the effects of CNM and 43 °C hyperthermia on ROS production. Furthermore, Annexin V staining indicated that CNM and hyperthermia treatment failed to induce apoptosis in A549 cells in the presence of NAC, suggesting the key role of ROS in the effects of this combination treatment (Figure 6b). Western blot analysis showed that when NAC blocked ROS generation, the effect of CNM plus hyperthermia co-treatment on levels of MAPKs phosphorylation (Figure 6c) and the cleavage of caspase-3 (Figure 6d) were interrupted.

## 3. Discussion

Lung cancer is the most responsible for cancer-associated death, causing approximately 1.6 million deaths per year. Although recent progress has been promising, major challenges remain [29]. This paper suggests a novel approach for NSCLC treatment using a less harmful natural compound, CNM, together with hyperthermia therapy.

CNM, a component of cinnamon, is considered a promising therapeutic agent for cancer treatment. Cinnamon is the dried bark of *Cinnamomum cassia*, used widely as an herbal medicine in traditional Korean medicine to improve blood circulation [30]. CNM is an organic compound comprising 90% of the essential oil of cinnamon, giving the scent and flavor [31]. Studies reported the anti-cancer effects of CNM in experimental models of cancer [5,6,7,8,9]. This study aimed to verify not only its anti-cancer effect in NSCLC but also to focus on combination therapy with hyperthermia, another potential treatment method that has been used to enhance the effects of anti-cancer agents.

Research has shown that a hyperthermal treatment in cancer patients can kill cancer cells and shrink tumors, while minimally affecting the normal tissues [32]. Although further evidence will be needed before hyperthermia can be recognized as a standard procedure, some trials have reported the beneficial effects of hyperthermia therapy for cancer treatment [33,34]. Furthermore, studies have shown that a combination treatment of hyperthermia with natural products may potentiate the anti-cancer effects [19,20,21]. Therefore, this study attempted to verify the combined effects of hyperthermia and CNM on the A549 NSCLC cell line. The results, including assessments of the cell viability, morphology, and migration, suggest that the CNM and hyperthermia co-treatment synergistically inhibited the proliferation of A549 cells (Figure 2).

Apoptosis is considered one of the most important targets for cancer management. The intrinsic pathway of apoptosis involves the response of the mitochondria. During apoptosis, cytochrome c is released from the mitochondria and binds with apoptotic protease activating factor-1 and ATP, then binds to pro-caspase-9 to form an apoptosome complex, which cleaves caspase-9 and in turn activates the cleavage of caspase-3 [22]. The results show that CNM and hyperthermia synergistically increased the cleavage of caspase-3 and 9 (Figure 3a), suggesting that the combination treatment enhanced the induction of apoptosis. Furthermore, intrinsic apoptosis, which is dependent on the balance between the pro-apoptotic and anti-apoptotic members of the Bcl-2 family members, such as Bcl-xL and Bcl-2 [22], was also suppressed by CNM and hyperthermia, as shown by the levels of Survivin, Bcl-xL, and Bcl-2 (Figure 3b). Mitochondrial membrane potential, one of the many indicators of the cell health profile, has been implicated as being associated in apoptotic cell death [35]. Further assays on Annexin V expression and mitochondrial membrane potential changes showed that CNM and hyperthermia co-treatment led to apoptosis of A549 cells (Figure 4a,b).

The eukaryotic cell cycle has four phases: G1, S, G2, and M phases. After DNA replication in the S phase, the cells continue to grow during the G2 phase and then enter the mitotic M phase, in which they divide into two daughter cells. A common feature most cancers share is disturbed apoptosis, which is caused by hyperactivated cell cycles [36]. Cyclin B1 and Cyclin D1 control mitosis, cell adhesion, and migration within the cell cycle; hence, they are associated with cancer cell development and metastasis [37]. In NSCLC cells, decrease in Cyclin B1 results in G2 phase cell cycle arrest to trigger apoptosis [38], whereas Cyclin D1 is also shown to be an important component during the arrest of the cell cycle [39]. Concomitantly, our results show that combination therapy with CNM and hyperthermia induced cell cycle arrest in A549 cells (Figure 4c). In addition, the metastatic potential of A549 cells was suppressed by the combination treatment. VEGF, the key molecule of angiogenesis [40], and MMP-2 and MMP-9, which are members of the metastasis-regulating MMP family [41], were decreased significantly by a co-treatment with CMN and hyperthermia (Figure 3d), indicating the beneficial effects of CNM and hyperthermia co-treatment in the improvement of metastasis.

HSP70 is a ubiquitous chaperone that controls a range of cellular processes, such as protein folding and maturation [42]. In particular, in response to thermal stress, HSP70 acts to protect cells. HSP70 also induces oncogenesis, proliferation, migration, and metastasis while suppressing apoptosis, specifically in cancer [43]. In the present study, while neither the sole normothermia treatment nor combination with CNM affected HSP70 expression, the hyperthermia treatment led to an increase in HSP70, suggesting activation of the defense mechanism against heat. However, combination with CNM inhibited this increase in HSP70, leading to the efficient induction of apoptosis. This suggests that CNM can potentiate the effect of hyperthermia by suppressing the defense mechanism against heat (Figure 3c).

The ROS signaling pathway is a widely accepted action mechanism of the apoptotic events in cancer cells by hyperthermia [10]. ROS are already a promising molecular target for the treatment of cancer [26] and are novel targets for herbal treatments [27]. Reports from hyperthermic chemotherapy revealed the definite involvement of ROS in hyperthermia treatment [44]. Moreover, the synergistic combination of hyperthermia with chemotherapy agents [45], nutritional support [46,47], or nature-derived materials, such as baicalin [48] and shikonin [49], involve ROS signaling. Similarly, the present results show that a co-treatment of CNM with 43 °C hyperthermia could also be a novel therapeutic approach towards NSCLC. Through a combination with CNM and hyperthermia, ROS signaling was induced in A549 cells, and MAPKs, the downstream targets of ROS, were also increased by the combination therapy of CNM and hyperthermia (Figure 5). The Akt pathway plays repressive roles in apoptosis via the canonical pathway [50]. On the other hand, several studies reported that Akt activation does not necessarily reduce apoptosis, but, in turn, remodels the sensitivity of cancer cells to metabolic stress [51,52,53]. In particular, when mitochondrial ROS are involved, Akt acts as the upstream channel that induces ROS signaling [54]. Moreover, the present results identify increased Akt phosphorylation along with induced ROS (Figure 5b). An additional study regarding a ROS scavenger work proved that ROS signaling was necessary for the apoptotic effects of CNM and hyperthermia combination treatment (Figure 6). These results may provide critical evidence for selecting ROS-targeted combination therapy of CNM and hyperthermia.

Overall, the results clearly show the apoptotic effect of combination therapy with CNM and hyperthermia treatment in A549 cells, which is dependent on the increase in ROS. This indicates the possible use of a combination treatment of CNM and hyperthermia as an effective approach for NSCLC treatment.

## 4. Materials and Methods

### 4.1. Reagents

CNM was purchased from Sigma-Aldrich (St. Louis, MO, USA) and prepared in dimethyl sulfoxide (DMSO) (Samchun Chem, Seoul, Korea). Anti-caspase-3, anti-heat shock protein (HSP) 70, anti-caspase-8, anti-caspase-9, anti-p-ERK (Thr202/Tyr204), anti-ERK, anti-p-p38 (Thr180/Tyr182), anti-p38, anti-p-JNK (Thr183/Tyr185), and anti-JNK antibodies were supplied by Cell Signaling Technology (Danvers, MA, USA). Anti-β-actin, anti-Bcl-2, anti-Bcl-xL, anti-Cyclin D1, anti-VEGF, anti-MMP-2, and anti-MMP-9 were obtained from Santa Cruz Biotechnology, Inc. (Dallas, TX, USA), and anti-cleaved caspase antibodies were acquired from GeneTex, Inc. (Irvine, CA, USA).

### 4.2. Cell Culture

The NSCLC cell line, A549 cells, were obtained from the Korean Cell Line Bank (Seoul, Korea) and maintained in DMEM medium supplemented with 10% fetal bovine serum (FBS) (Gibco, Grand Island, NY, USA) and 1% penicillin-strep (Gibco, Grand Island, NY, USA) at 37 °C in an incubator with humidified air containing 5% CO₂, as described elsewhere [55].

### 4.3. Hyperthermia Treatment

The A549 cells were seeded in a 6-well plate (3 × 10^5^ cells/well), suspended in 3 mL of media, followed by immersion in a temperature-controlled water bath at 37 or 43 °C for 30 min. CNM at the indicated concentrations (150 and 200 μM) was added 60 min before the hyperthermia treatment.

### 4.4. MTT Assay

An MTT assay was used to evaluate the cell viability after exposure to CNM with hyperthermia. Briefly, A549 cells were seeded in 96-well plates (1.5 × 10⁴ cells/mL) and allowed to adhere overnight. Various concentrations of CNM (150 and 200 μM) were added, and the plates were incubated at 37 °C for 1 h in a humidified atmosphere containing 5% CO_2_. The resulting plates were then immersed in a temperature-controlled water bath at 37, 42, or 43 °C for 30 min. After 48 h of incubation at 37 °C, 5% CO_2_, an MTT solution (AMRESCO, Solon, OH, USA) was added and incubated for an additional 2 h. The culture medium was discarded, and the cells were lysed in 100 μL of DMSO. The absorbance was measured using an automated spectrophotometric plate reader at 570 nm. The cell viability was normalized as the relative percentages in comparison with the untreated controls. The synergistic effects of the CNM and hyperthermia combination were determined based on the combination index, which was calculated using CompuSyn software ver. 1.0 (ComboSyn, Inc., Paramus, NJ, USA).

### 4.5. Trypan Blue Staining

Live/dead cell ratio was determined by Trypan blue staining. Briefly, A549 cells were seeded in 6-well plate (3 × 10^5^ cells/well) and treated with CNM for 1 h and hyperthermia for 30 min. After 24 h of post-treatment incubation, cells were harvested, diluted in PBS (1:4), stained with Trypan blue (Sigma-Aldrich, St. Louis, MO, USA), then viable cells and formed colonies were counted. Cell survival rate was calculated as follows:

(1) $$Cell\;survival\;rate\;\left(\%\right)=\frac{Viable\;cell\;count}{Total\;cell\;count}\;\times \;100\;\;$$

### 4.6. Morphology Assay

A549 cells were seeded in a six-well plate (3 × 10^5^ cells/well), treated with 150 or 200 μM of CNM for 1 h, and incubated at 37 or 43 °C for 30 min. After 24 h, the cells were visualized, and representative images were obtained under a regular optical microscope (CX-40, Olympus, Tokyo, Japan).

### 4.7. Wound healing Assay

The cells were seeded in a six-well plate at a density of 5 × 10^5^ cells and cultured at 37 °C. After confluence, a thin scratch was produced in each well with a yellow pipette tip. The culture medium was removed after 24 h, and representative images were obtained under a regular optical microscope (CX-40, Olympus, Tokyo, Japan).

### 4.8. Clonogenic Assay

Four hundred cells/well were seeded in a six-well plate and incubated overnight. The following day, the cells were treated with 200 μM CNM for 1 h and incubated at 37 or 43 °C for 30 min for the hyperthermia treatment. After two weeks, the cells were stained with a crystal violet (Sigma-Aldrich, St. Louis, MO, USA) solution at RT for 10 min and washed with PBS. Images of the colonies were obtained under a regular optical microscope (CX-40, Olympus, Tokyo, Japan).

### 4.9. Western Blot Analysis

Western blot analysis was performed as described elsewhere [56]. Briefly, after the protein concentrations from isolated A549 cells were determined, equal amounts of lysates resolved by sodium dodecyl sulfate (SDS)-polyacrylamide gel electrophoresis were transferred to a polyvinylidene difluoride membrane, and the membrane was blocked with 1x TBS containing 0.1% Tween 20 and 5% skim milk at RT. The membranes were incubated overnight at 4 °C with the respective primary antibodies followed by washing and incubation (1 h, RT) with diluted horseradish peroxidase-conjugated anti-rabbit or anti-mouse IgG antibodies (Santa Cruz Biotechnology, Inc., Dallas, TX, USA). The immunoblot signals were detected using an enhanced chemiluminescence kit (EMD Merck Millipore, Billerica, MA, USA). Membranes were incubated in stripping buffer containing 2% SDS, 62.5 mM Tris-HCl (pH 6.8) and 0.7% mercaptoethanol in D.W. at RT for 30 min to detach antibodies to confirm the total forms of ERK, p38, JNK and Akt after detecting their phosphorylation forms.

### 4.10. Apoptosis Assay

A Muse^®^ Annexin V and Dead cell kit (Part Number: MCH100105) (EMD Merck Millipore, Billerica, MA, USA) was used to measure the ratio of apoptosis. A549 cells were seeded in 6-well plates at a density of 3 × 10^5^ cells/well. Twenty-four hours after treatment with CNM and hyperthermia, A549 cells were collected, and pellets were subjected to an Annexin V and 7-amino-actinomycin D (7-AAD) staining according to the manufacturer’s instructions. After incubation, the cells were analyzed using the Muse^®^ Cell Analyzer (EMD Merck Millipore, Billerica, MA, USA).

### 4.11. Cell Cycle Analysis

A549 cells (3 × 10^5^ cells/well) in six-well plates were exposed to co-treatments for 24 h, and the cell cycle phase was analyzed. The cells were then collected, fixed overnight in 70% ice-cold EtOH, washed with PBS, and resuspended in PBS containing 1 mg/mL PI, 10 mg/mL RNase A in a dark room for 10 min. The cell cycle was determined using a Muse^®^ Cell Analyzer (EMD Merck Millipore, Billerica, MA, USA).

### 4.12. Analysis of Reactive Oxygen Species (ROS)

The levels of ROS generation were measured using a ROS assay kit (Part Number: MCH100111) (EMD Merck Millipore, Billerica, MA, USA). Twenty-four hours after the co-treatment of CNM and hyperthermia, the A549 cells were treated with an oxidative stress working solution and incubated for 30 min at 37 °C. The ROS levels were analyzed using the Muse^®^ Cell Analyzer (EMD Merck Millipore, Billerica, MA, USA). The cells were treated with NAC, the ROS inhibitor [57], for 1.5 h before treatment with CNM and hyperthermia.

### 4.13. Statistical Analysis

All numeric values are represented as the mean ± SD. The statistical significance of the data compared to the untreated control was determined using Student’s unpaired *t*-test * *p* < 0.05, ** *p* < 0.01 and *** *p* < 0.001 were considered significant.

## Supplementary Materials

Supplementary materials can be found at https://www.mdpi.com/1422-0067/21/17/6229/s1, Figure S1: Effect of CNM and hyperthermia combination on Cyclin B1 expression in A549 cells.

## Abbreviations

NSCLC	non-small cell lung cancer
CNM	cinnamaldehyde
Bcl	B-cell lymphoma
HSP70	heat shock protein 70
VEGF	vascular endothelial growth factor
MMP	matrix metallopeptidase
ROS	reactive oxygen species
MAPK	mitogen-activated protein kinase
ERK	extracellular signal-regulated kinase
JNK	c-Jun NH2-terminal kinase
NAC	N-acetylcysteine
DMSO	dimethyl sulfoxide
FBS	fetal bovine serum
