# Supplementary material for: Combination Therapy with Cinnamaldehyde and Hyperthermia Induces Apoptosis of A549 Non-Small Cell Lung Carcinoma Cells via Regulation of Reactive Oxygen Species and Mitogen-Activated Protein Kinase Family
Source: Int J Mol Sci. 2020 Aug 28;21(17):6229. doi: 10.3390/ijms21176229 (PMC7504317; doi:10.3390/ijms21176229)
Supplement: Supplementary file 1 [file ijms-21-06229-s001.pdf]

Supplementary Material

Combination Therapy with Cinnamaldehyde and Hyperthermia Induces Apoptosis of A549 Non-Small Cell Lung Carcinoma Cells via Regulation of Reactive Oxygen Species and Mitogen-Activated Protein Kinase Family

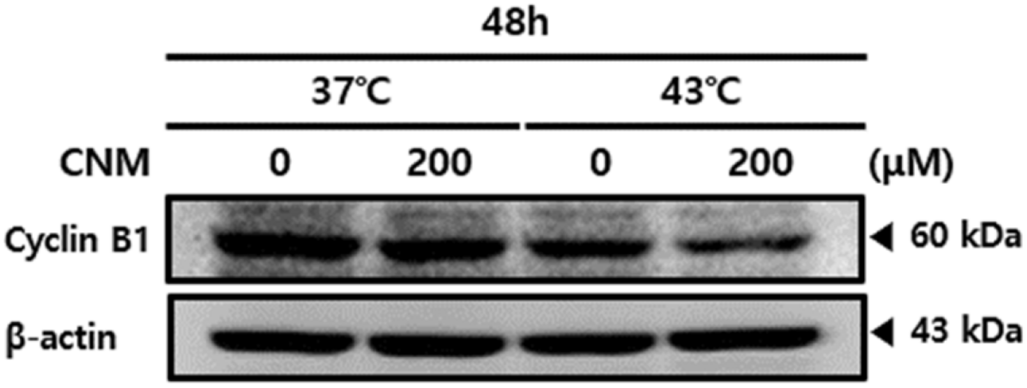

Figure S1. Effect of CNM and hyperthermia combination on Cyclin B1 expression in A549 cells.
